# A novel velocity band energy workflow for fiber-optic DAS interpretation and multiphase flow characterization

**DOI:** 10.1038/s41598-023-42211-0

**Published:** 2023-09-13

**Authors:** Gerald. K. Ekechukwu, Jyotsna Sharma, Michael J. William

**Affiliations:** 1 Louisiana State University, Baton Rouge, USA; 2 Schlumberger Cambridge Research, Madingley Road, Cambridge, CB3 0EL United Kingdom

**Keywords:** Fibre optics and optical communications, Optical sensors, Geophysics, Engineering

## Abstract

Distributed fiber-optic sensing continues to gain widespread adoption in the energy industry because of the numerous benefits it offers for real-time surface and subsurface monitoring of pipelines, wellbores, reservoirs, and storage infrastructure. In this study, we introduce a novel workflow to analyze optical fiber-based distributed acoustic sensor (DAS) data, which takes into account the speed of sound for a certain phase to filter the acoustic energy or signal contributed by that phase. This information is then utilized for the characterization of multiphase flow. The application of the proposed velocity band energy (VBE) workflow is demonstrated using a dataset acquired in a 5163-ft-deep wellbore, for estimating gas void fraction and real-time gas–liquid interface tracking across the length of the well. The workflow utilizes a series of signal processing and conditioning steps that aim to reduce noise and enhance the signals of interest. The insights from the new methodology will further assist in validating DAS-based flow monitoring algorithms, leak detection and quantification, and reservoir characterization.

## Introduction

In recent years, the deployment of distributed acoustic sensors (DAS) has continued to gain prominence in the energy industry for real-time monitoring of surface and subsurface infrastructure, dynamic processes, and geophysical evaluation. Some of the most common applications include flow profiling^1–5^, fracture monitoring^6,7^, leak detections^8^, structural health monitoring^9,10^, and vertical seismic profiling^11–13^. DAS typically uses the phase shifts of the Rayleigh component of the backscattered light propagating through the fiber core^14,15^. The spatiotemporal DAS vibration data are extracted in the form of a two-dimensional (2D) time-depth matrix, where each data trace (columns) corresponds to a single laser pulse traveling down the fiber cable and back to the interrogator, at a given time instance.

Multiphase flow is defined as the simultaneous flow of more than one phase through a channel^16^ such as porous media or tubulars such as pipelines and wells. The multiphase flow in pipeline is characterized by flow regime, flow rate, and fluid fractions. Gas void fraction is the volume occupied by the gas in a multiphase flow scenario. It is an important parameter for two-phase flow calculations such as mixture viscosity, mixture density, and pressure drop calculations. Many experimental studies and several correlations have been proposed to estimate this essential parameter in two-phase flow^17,18^. Many of these correlations are suited for specific directions (either upward or downward flow) and flow regimes. In addition, electrical methods, radioactive, ultrasonic, thermal, and differential pressure methods^19^ have all been proposed as techniques for measuring gas void fraction in gas–liquid flows. However, these conventional techniques have several limitations. For example, downhole multiphase flow meters or gauges only provide information at discrete locations, rather than simultaneously across the entire wellbore interval. Interface tracking detection using wireline logging techniques can be expensive and results in downtime for production. The conductivity method obtains void fraction based on the conductivity between the different phases. The difference in water salinity in oil wells however leads to measurement errors^19^. Safety, cost concerns, and severe intensity attenuation have limited the use of radioactive methods. Strong scattering of the ultrasound signals by the gas phase in the two-phase mixture also limits the use of the ultrasound method^19^. Another challenge for the in-well measurement techniques is withstanding high pressure and high temperature environments that are prevalent downhole.

Due to the above limitations and challenge, the use of DAS for fluid flow characterization has lately gained popularity. One of the biggest advantages of fiber-optic sensors as compared to conventional sensors and gauges, is the ability to provide spatially and temporally continuous information across the length of the fiber, without the need of any electronics along the optical path. DAS could detect vibrations induced by flow and the speed of sound (SOS) changes that are encountered during multiphase scenarios. An increasing number of studies are being carried out to understand the multiphase flow in pipelines, however in many of them, the experimental procedures were carried out in flow loops. While these studies are crucial, more in-well studies are needed to better understand the multiphase flow dynamics in at well scale. This is especially true because the bubble rise velocity in multiphase flow is a strong function of the annular diameter as observed by Rader et al.^20^. To this effect, a series of gas–water flow tests have been conducted in an experimental well at LSU since 2019 to study multiphase flow dynamics using distributed fiber-optic sensors. The effect of different gas volumes, liquid circulation rates, pressures, and injection methods on flow behavior were investigated and the fiber-optic DAS and distributed temperature sensor (DTS) measurements. The DAS and DTS data were analyzed using a variety of signal processing techniques, including, frequency band energy (FBE) extraction, frequency-wavenumber (or F-K) transform, energy spectrums, and gradient plots. Some of these analysis and results pertaining to gas velocity calculation, flow rate measurement, and pressure estimation have been published in our earlier publications^21–24^. However, none of them included the estimation of gas void fraction and interface tracking, which is the focus of the current study.

The SOS approach has been successfully used by some authors for multiphase flow characterization, specifically for the estimation of phase fractions. For example, Finfer et al.^25,26^, by wrapping a continuous length of fiber helically around a short section of a pipe in their flow loop experiment, used a DAS system to measure the dynamic radial strain (hoop strain) exerted along the short section of pipe. They were able to obtain the SOS directly on the F-K domain plot and the flow velocity via eddy tracking on the time domain plot. However, challenges have been reported in using SOS for phase fraction estimations for in-well multiphase characterization studies. For instance, during the early stages of this study, we were unable to obtain plausible SOS values directly on the F-K domain plots. The SOS values estimated were similar to those reported by Paleja et al.^3^, who in their experimental study, found that the SOS in an air–water mixture to be 10–20 m/s, which was less than that of water (1450 m/s) or air (300 m/s), hence it was not possible to estimate the gas fraction using those values. The ability to use the eddy-tracking method (enabled by the helical wrapping) requires spatial resolution with an order of magnitude comparable to the pipe diameter^25^. However, many wells including the test well used in this study is installed with a straight fiber configuration, hence we continued to explore how to obtain realistic SOS using additional approaches. Other previous studies, e.g., Johannessen et al.^27^, Naldrett et al.^28^ and Bukhamsin et al.^29^ that have also explored the SOS method using straight fiber configuration, had inflow control devices (ICDs) in their wellbore that created restrictions in the wellbore. Flow-through ICDs created enhanced perturbations that would be easier to detect using DAS. However, not all wells are instrumented with ICDs, including the test well used in this study^30^. Since there are no restrictions in the wellbore, the self-generating pressure waves due to fluid flow may not be strong enough for implementing the SOS workflow demonstrated by Bukhamsin et al.^29^ for phase fraction estimation.

To address the multiphase flow characterization challenge described above, we introduce a new approach called the velocity band energy or VBE workflow for processing DAS vibration data. The VBE method considers the SOS of a phase in a multiphase flow to extract the phase-specific acoustic energy or signal. This helps in the estimation of the flow characteristics of that phase. Because DAS data can be voluminous (up to multiple terabytes per day for wellbore monitoring), the VBE approach also has the practical benefit of compressing the data without losing valuable information. Furthermore, since DAS provides spatially continuous information across the entire length of the fiber, the VBE workflow has the potential to provide gas void fraction simultaneously and continuously across the length of the installed fiber. This approach is flow-regime agnostic and suited for either upward and downward vertical or inclined flows as well as in both small and large diameter pipes. This method takes into account the SOS for a certain phase to filter the acoustic energy or signal contributed by that phase in a multiphase medium. We demonstrate the usefulness of the VBE workflow for two important applications related to multiphase flow characterization: identifying gas–liquid interfaces and estimating gas void fraction in mixtures in well-scale data.

## Experimental procedure and analysis methodology

### Test well setup

**Figure 1:**
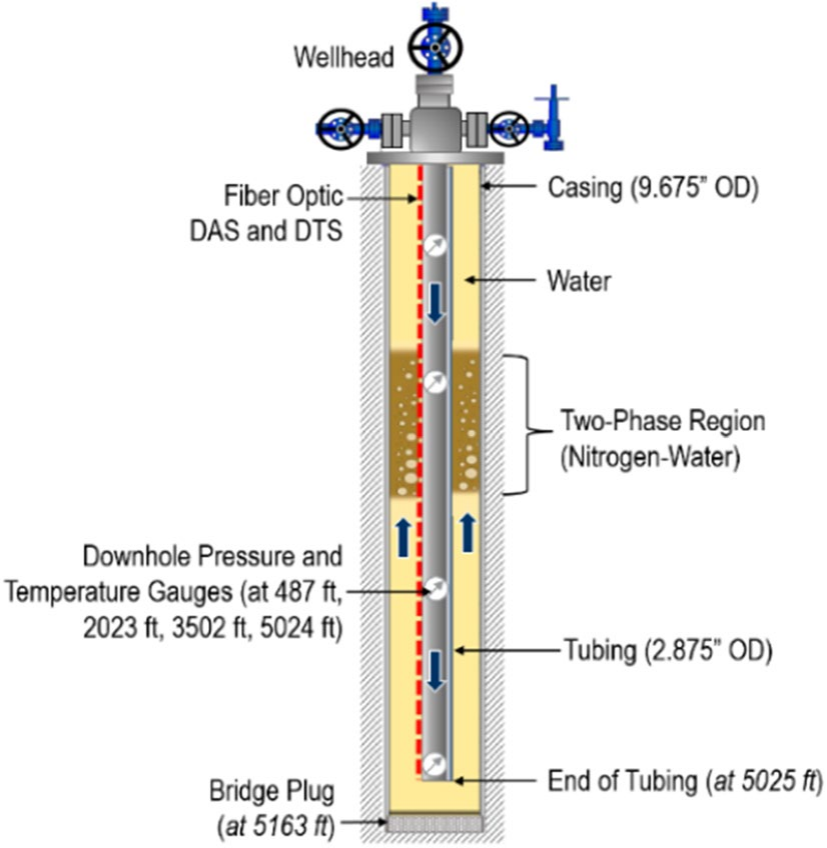
Schematic of the test well used in the study.

The data analyzed in this study was acquired during two-phase flow tests conducted in a full-scale 5163-ft-deep cased wellbore (Fig. 1), located in the Petroleum Engineering Research, Training, and Testing (PERTT) lab facility at Louisiana State University. The test well is instrumented with four downhole pressure and temperature gauges located at 487 ft, 2023 ft, 3502 ft, and 5025 ft depths. A quarter-inch control line carrying optical fibers is attached to the outside of the production tubing using steel clamps. The measurement parameters for the distributed acoustic sensor (DAS), as well as the properties of the optical fiber installed in the test well for DAS acquisition are summarized in Table 1.

**Table 1:** DAS acquisition parameters and optical fiber specifications.

	DAS
Fiber specifications	
Hermetic coating	Carbon
Coating	Polyimide
Core	Silica
Optical mode	Single mode
Core diameter (µm)	10 ± 0.5
Clad diameter (µm)	125 ± 2
Coating diameter (µm)	155 ± 2
Acquisition parameters	
Spatial resolution (m)	1.5
Sampling interval (m)	0.77
Recording length (s)	10
Acquistion frequency (Hz)	10,000

For the use case presented in this paper, the wellbore was initially filled with water (in both the tubing and the annulus). Then, a fixed volume (15 barrels or bbl) of nitrogen gas was injected at a constant rate down the tubing, with the surface backpressure set at 200 psi. During the gas injection, the volume of water being displaced by the gas is recorded every minute. When the cumulative returning volume reaches the pre-defined volume (15 bbl in this case), the gas injection was stopped. Water is then injected or bullheaded down the tubing at 300 GPM to displace the gas to the bottom of the well. Once the gas slug is at the bottom of the tubing, circulation is stopped and the gas is allowed to rise to the surface through the annulus. The pressures and temperatures, as well as DAS data were all recorded in real time during the experiment. The blue arrows in Fig. 1 indicate the direction of the gas movement injected down the tubing and then bubbling up to the surface through the annulus as a two-phase (nitrogen-water) mixture.

### Velocity band energy (VBE) workflow

**Figure 2:**
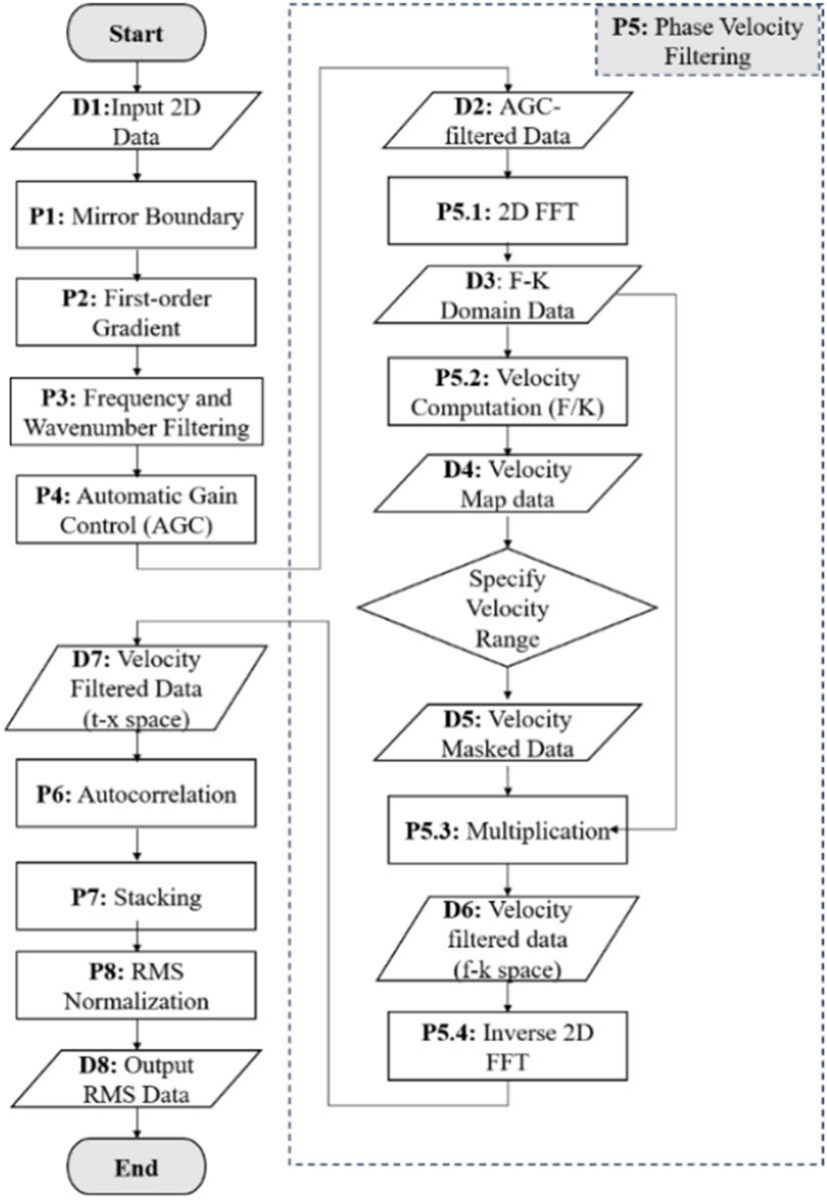
Illustration of the steps involved in the VBE workflow.

The steps involved in the VBE workflow are shown in Fig. 2 and described in detail in this section. The underlying computations involved in the workflow were coded using the Python programming language. Each step in the workflow diagram is numbered with the processes/computation steps represented by rectangles are prefixed with “P”, while the input/output/intermediary data points are represented with trapezoids and prefixed with “D”. The workflow starts with a representative 2D (time-depth) DAS vibration input data (**D1**) on which a series of signal-processing steps are implemented, which are aimed at reducing the noise in the data and enhancing the signal of interest (which is the gas signature in the case studies presented). Each of these steps are described in this section.

The mirror boundary condition (**P1**) is first applied to remove boundary artifacts on the input data. In image or 2D-data analysis, boundary value problems usually lead to ring or circular artifacts in the processed data if it is not properly handled. Various extrapolation techniques have been proposed to extend the continuity of the data such as using zero Dirichlet, periodic, and reflective or mirror boundary conditions (also called Neumann or symmetric)^31^. All of these were explored, however, the mirror boundary condition approach gave the best result in our case. For the Neumann boundary condition, it is assumed that the data outside of the data of interest, *f*, are a reflection of the data inside *f*. It can be mathematically represented by Eq. (1)^31^as follows:

1 $$\left\{\begin{array}{l}{f}_{0}= {f}_{1}\\ .\\ .\\ .\\ {f}_{-m+1}= {f}_{m}\\ \end{array}\right. \; \mathrm{and} \; \left\{\begin{array}{l}{f}_{n+1}= {f}_{n}\\ .\\ .\\ .\\ {f}_{m+m}= {f}_{n-m+1}\\ \end{array}\right.$$

where ${f}_{1}$ to ${f}_{m}$ represent the top *m* rows inside of the data (*f*) and ${f}_{n-m+1}$ to ${f}_{n}$ represent the bottom *m* rows inside of the data.

Next, a first order gradient filter (**P2**) is applied on the 2D data to remove the horizontal bands that were observed in the test well data used in this study, which are a result of the nonuniform coupling of the fiber to the production tubing. (This step may be skipped based on the specific data observations.) This is followed by frequency and wavenumber (or F-K) filtering (**P3**). F-K filtering is a popular seismic processing technique for separating useful signals from noise (such as ground rolls or multiples) on the basis of dip or apparent velocity differences^32^. F-K filtering has also been used in DAS processing to detect the acoustic energy propagation and the frequency-dependent attenuation associated with distance^33^. In this study, the F-K filtering was computed as frequency filtering followed by wavenumber filtering steps. For the frequency filtering, a high-pass Butterworth filter is applied in the time direction to remove the DC noise (i.e. at zero frequency) and pump noise present in the signal. The motivation of the wavenumber filtering is to optimize the visualization of the signal wavelengths of interest, which in our case was the tracking of the gas–water mixture region for detecting the interface and gas void fraction. To achieve this, a Butterworth filter is applied in the depth direction to ensure that the long wavelengths are removed from the data to optimize the visualization of the extended gas bubble in the fluid mixture. The Butterworth filter is a time or depth-domain filter in which the frequency response is as flat as possible in the bandpass.

The F-K filtering is followed by the application of the automatic gain control (AGC) (**P4**). The AGC is another commonly used seismic processing technique^34^, which is applied to enhance the weak signals in the data to improve the signal-to-noise ratio (SNR). The weak signals are introduced by the gas bubbles which are more attenuating compared to the flowing water. The AGC computation involves multiplying the DAS data with a scaling factor $g\left(x\right)$ which is estimated in Eq. (2) as the inverse of the root mean square of all the DAS data at that depth for each time window considered as:

2 $$g\left(x\right)= \frac{1}{\sqrt{\frac{1}{N}{\sum }_{1}^{N}{A}_{i}}}$$

where ${A}_{i}$ is the DAS data in a given row in the 2D matrix, and $N$ is the total number of samples in each depth in the recording time window, which is 10 s in our case.

The next step in the VBE workflow is the phase velocity filtering step (**P5**). This involves a series of sub-steps as illustrated in the dash rectangle of Fig. 2. The phase velocity filtering step takes the AGC-filtered data (**D2**) as an input. Two dimensional fast Fourier transform (2D FFT) as shown in Eq. (3) (**P5.1**) is then applied to convert the AGC-processed data to the frequency-wavenumber or F-K domain (**D3**).

3 $$F\left(f,k\right)=\iint f\left(t,x\right){e}^{-\left(kx-2\pi ft\right)}dxdt$$

The properties of the F-K domain data are used to create 2D frequency and 2D wavenumber matrices from which a velocity map (**D4**) is created by taking the ratio (**P5.2**) of the frequency to the wavenumber for each corresponding cell in the 2D frequency and 2D wavenumber matrices. A user-defined velocity range of interest is then specified to create a velocity mask. The velocity range selected depends on the fluids of interest. In our use case, we are interested in gas (nitrogen) and water mixtures, hence, for the detection of gas–water two-phase region a velocity range of 900 to 1200 ft/s is specified based on the speed of sound in nitrogen-water mixture^35^. Similarly, for the detection of the single-phase water region, a velocity range of 4900 to 5200 ft/s is specified^35^, corresponding to the speed of sound in water. The velocity map is then multiplied with the resulting velocity mask to obtain the velocity-masked data (**D5**). The F-K domain data (**D3**) is then multiplied with the velocity masked data (**D5**) to obtain a velocity-filtered data (**D6**) (in the F-K space). Lastly, the inverse 2D fast Fourier transform (**P5.4**) is applied to present the data in the time–space domain. The phase velocity-filtered data is further passed through an autocorrelation function step (**P6**) of the VBE workflow. The autocorrelation function is used to obtain the degree of similarity of a time series with itself, which helps to obtain the periodic information embedded in the data^36^. The autocorrelation of an analytical function $x(t)$ is given:

4 $${R}_{xx}\left(\tau \right)= {\int }_{-\infty }^{\infty }x\left(t\right)x\left(t+\tau \right)dt= {\int }_{-\infty }^{\infty }x\left(t-\tau \right)x\left(t\right)dt$$

**Figure 3:**
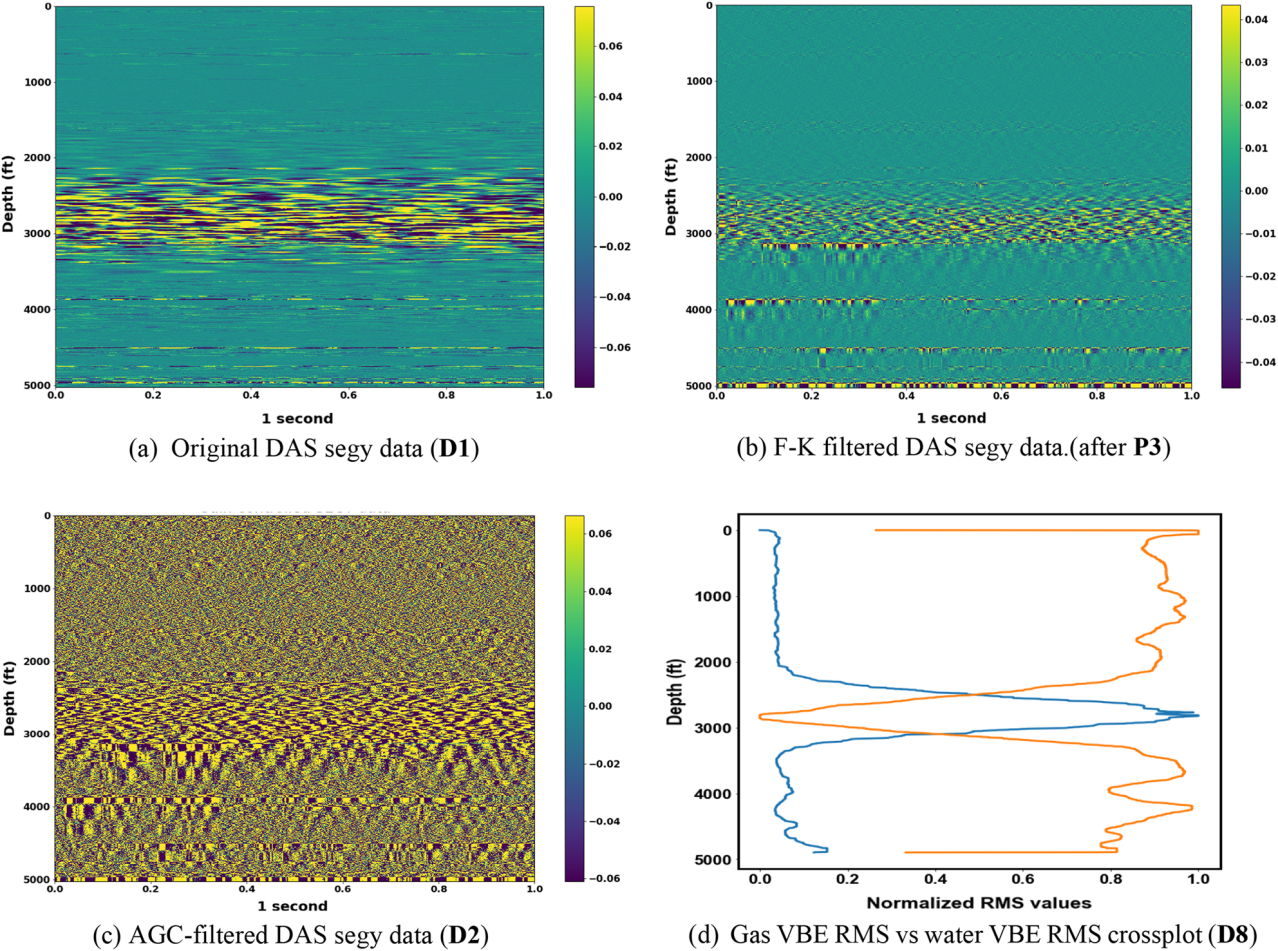
Original DAS data and illustration of a some of the steps in the VBE workflow.

Calculating the autocorrelation of the signal $x(t)$ is the same as the square of the amplitude spectrum in the frequency domain. In other words, taking the power spectrum of $x(t)$ in the frequency domain is another way to compute the autocorrelation function.

5 $$\mathfrak{I}\left\{{R}_{xx}\left(\tau \right)\right\}={R}_{xx}\left(\omega \right)= X\left(\omega \right).X\left(\omega \right)={\left|X\left(\omega \right)\right|}^{2}$$

where $\omega$ is the angular frequency. In this work, the autocorrelation was computed at each depth in the frequency domain, and then the inverse Fourier transform was applied to convert the result back to the time–space domain, which is then stacked (**P7**) before the root-mean-square (RMS) is computed. The RMS values are then normalized (**D8**) using the minimum–maximum approach (**P8**) to enhance phase detection. An illustration of the original DAS data and the results from some of the steps of the VBE workflow are presented in Fig. 3. In summary, using the VBE workflow, the speed of sound in the fluid or mixture can be obtained by manually measuring the slopes of the upgoing and down going waves after the F-K computation step (**P3**). Additionally, the data at the end of the workflow (**D8**) can then be used to track the fluid–fluid interface as described in the next section.

## Applications of VBE workflow

### Case study 1: gas–liquid interface tracking

**Figure 4:**
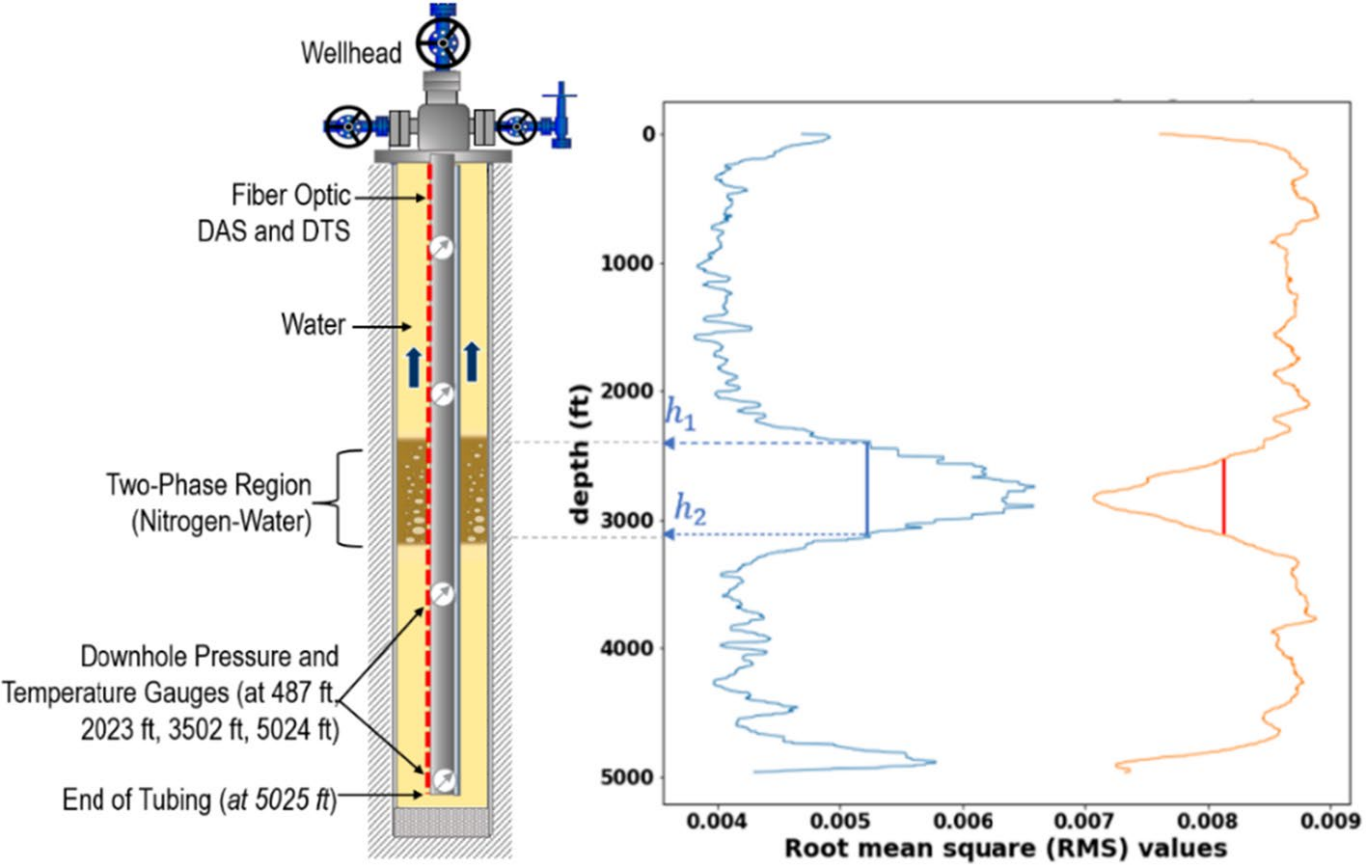
Gas–water mixture interface tracking using FWHM (the blue curve is the VBE RMS for the gas–water mixture while the orange curve is the VBE RMS for the water phase). The RMS plots are obtained at the finl step (**D8**) of the workflow.

Figure 4 shows two normalized VBE RMS traces obtained from the VBE workflow using the same input DAS data. For the blue curve, a velocity mask corresponding to the speed of sound in nitrogen-water mixture (900 to 1200 ft/s) is applied, while for the orange curve a velocity mask corresponding to the speed of sound in single-phase water (4900 to 5200 ft/s) is applied. A high VBE RMS value for the water VBE RMS indicates the presence of single-phase water, while a high value for the gas–water VBE RMS indicates the presence of gas–water (two-phase) region. The interface between the single-phase water and the gas–water mixture regions as well as the height of the two-phase gas–water region can be deduced based on the full width at half maximum (FWHM)^37^ using the VBEs corresponding to water and gas–water mixtures. The FWHM is the difference between the two values of a variable (in our case, height) at which the other variable in a plot (in our case, RMS) is equal to half of its maximum. As illustrated in Fig. 4, h1 and h2 approximate the depths of the two interfaces and the gas–water (two-phase region) height can be estimated as *h*_*2*_ minus *h*_*1*_. This corresponds to the moment when the gas–water mixture is between gauges at 2023 ft and 3502 ft, as shown in the schematic. From Fig. 3, the gas–water column height can be approximated as 774 ft.

**Figure 5:**
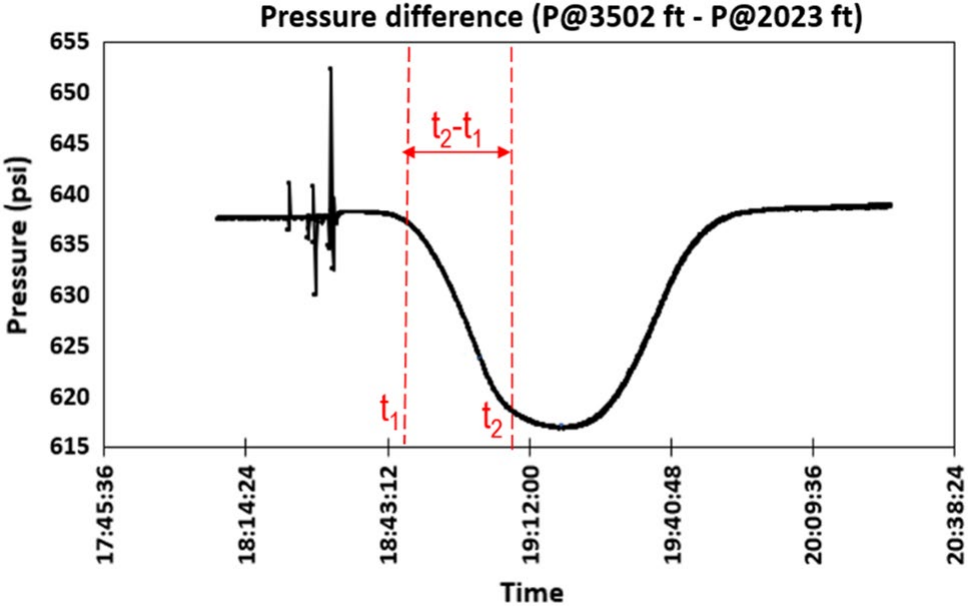
Pressure difference between the gauges at 3502 ft and 2023 ft as a function of time.

To validate the result presented in Fig. 4, the pressure difference between gauges at 3502 ft and 2023 ft was analyzed, as shown in Fig. 5. The time instance when the pressure difference between these gauges first starts to drop (*t*_*1*_) indicates the gas–water interface first crossing that gauge location. Eventually, the pressure difference plateaus (at time *t*_*2*_), which indicates that the gas–water mixture region has completely crossed the gauge at 3502 ft and is now between gauges at 2023 and 3205 ft. To estimate the height of the gas–water mixture region, we take the time difference (*t*_*2*_ minus *t*_*1*_) and multiply that with the average gas rise velocity estimated experimentally and validated using numerical simulations by Sharma et al.^24^ for this test as 0.5 ft/s. This gives an average column height of 772 ft, which is in good agreement with the estimate using the VBE workflow.

### Case study 2: Gas void fraction estimation

Gas void fraction is the volume occupied by the gas in a multiphase flow scenario. It is an important parameter for two-phase flow calculations such as mixture viscosity, mixture density, and pressure drop calculations. Here, we present a novel application of the VBE workflow for real-time gas void fraction estimation. Because DAS provides spatially continuous information across the entire length of the fiber, the VBE workflow has the potential to give us gas void fraction simultaneously at different depth intervals. This approach is flow-regime agnostic and suited for either upward and downward vertical or inclined flows, as well as in both small and large diameter pipes. The fundamental principle employed in this method is the estimation of the speed of sound in a multiphase medium.

The speed of sound in a gas–water mixture as a function of the gas void fraction, *α*_*g*_, can be described using Eq. (4), as proposed by Chaudhuri et al.^38^ and Bukhamsin et al.^29^.

6 $${c}_{m}= {\left\{\left[ {\alpha }_{g}{\rho }_{g}+\left(1-{\alpha }_{g}\right){\rho }_{w}\right]\left[\frac{{\alpha }_{g}}{{K}_{g}}+\frac{\left(1-{\alpha }_{g}\right)}{{K}_{w}}+ \frac{d}{Et}\right]\right\}}^{-\frac{1}{2}}$$

The gas and water moduli can be substituted as ${K}_{g}= {c}_{g}^{2}{\rho }_{g}$ and ${K}_{w}= {c}_{w}^{2}{\rho }_{w}$, respectively. The gas void fraction, ${\alpha }_{g}$, can then be calculated by solving the quadratic equation

7a $${\alpha }_{g}= \frac{-B \pm \sqrt{{B}^{2}-4AC}}{2A}$$

where the coefficients are defined as:

7b $$A= \left(\frac{1}{{c}_{g}^{2}}- \frac{{\rho }_{g}}{{\rho }_{w}{c}_{w}^{2}}\right)\left(1-\frac{{\rho }_{w}}{{\rho }_{g}}\right)$$

7c $$B= \frac{{\rho }_{g}}{{\rho }_{w}{c}_{w}^{2}}+ \frac{{\rho }_{w}}{{\rho }_{g}{c}_{g}^{2}}-\frac{2}{{c}_{w}^{2}}+\frac{{\rho }_{g}d}{tE}-\frac{d}{tE}$$

7d $$C=\frac{1}{{c}_{w}^{2}}-\frac{1}{{c}_{m}^{2}}+\frac{{\rho }_{w}d}{tE}$$

In the above equations*, c*_*g*_*, c*_*w*_, and *c*_*m*_ are the speed of sound in gas (nitrogen), water, and gas–water mixture, respectively, *K* is the bulk modulus of the medium, *ρ*_*g*_ and *ρ*_*w*_ are the gas and water phase densities, *d* is the pipe diameter, *t* is the pipe wall thickness, and E is the Young’s modulus. The speed of sound in fluid medium is also dependent on the pressures and temperatures because they affect the fluid properties.

**Figure 6:**
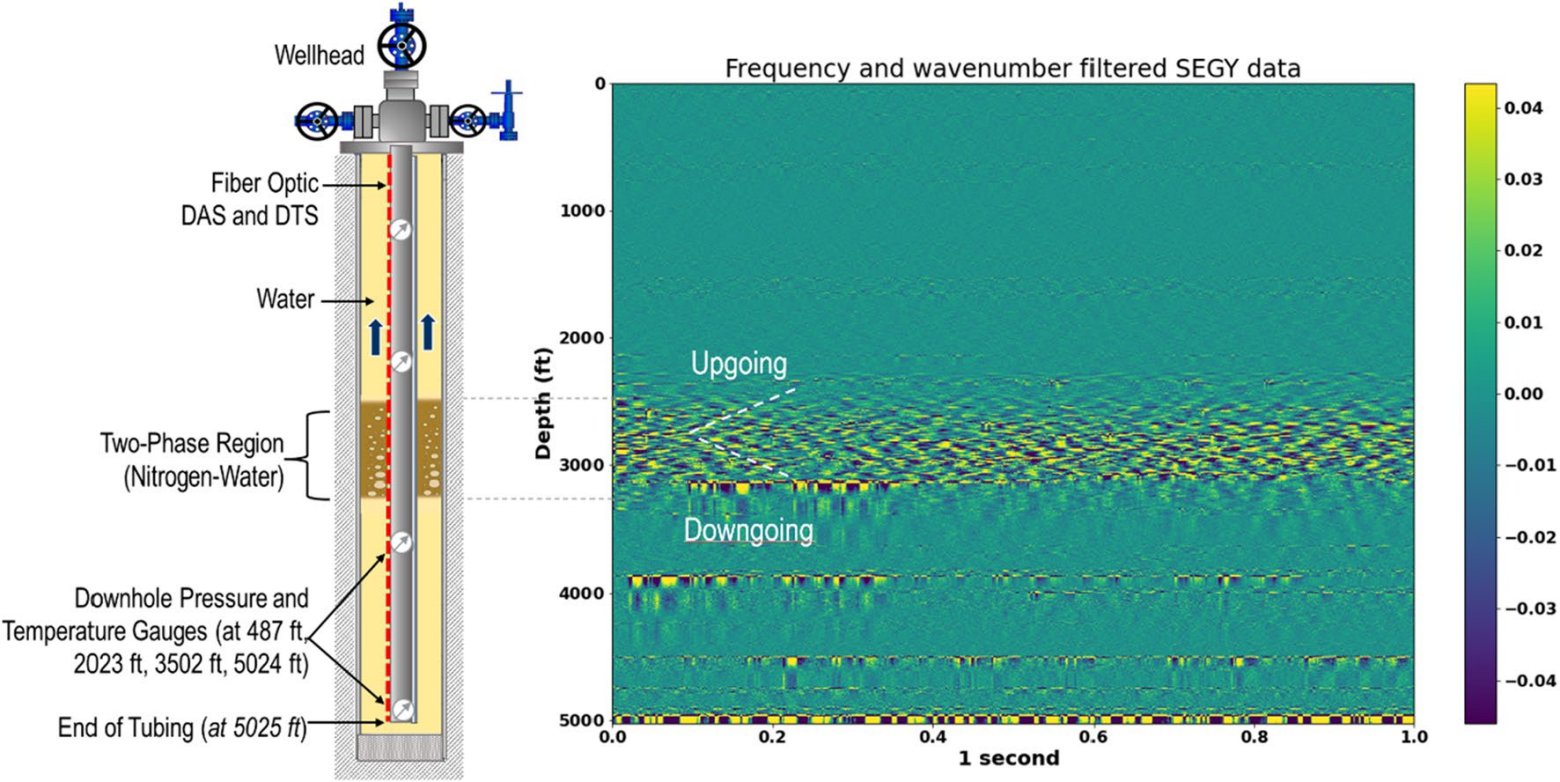
The upgoing and downgoing sound waves identified from the frequency-wavenumber filtered DAS data obtained from the VBE workflow (after step **P3**).

From the VBE workflow applied to the DAS data, the speed of sound in the gas–water mixture *(c*_*m*_*)* can be directly estimated by calculating the average of the different downgoing, and upgoing sound waves (slopes) identified in the F-K filtered plot obtained from the VBE workflow. Figure 6 illustrates this approach, where the white dashed lines highlight the upgoing and downgoing sound waves. Based on this approach, from Fig. 6, *c*_*m*_ is estimated as 1680 ft/s (based on the average slopes of several upgoing and down going waves that were manually measured) and using Eq. (7), the gas void fraction at this instance was estimated as 0.07.

**Figure 7:**
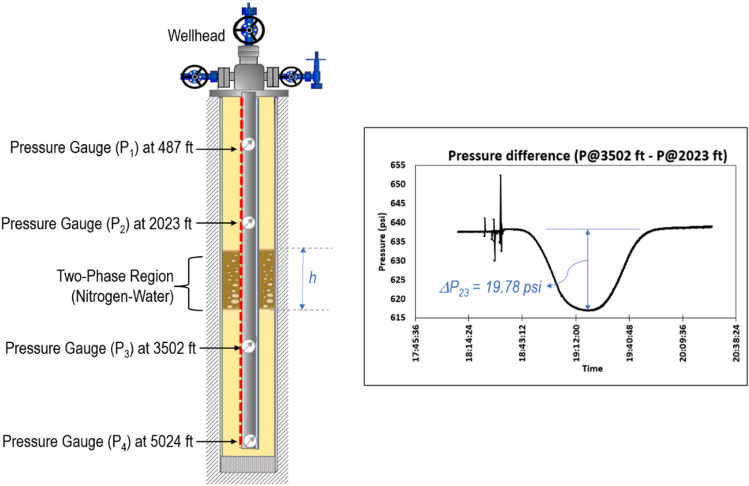
Schematics showing the gas rise in the annulus of the well.

To validate the gas void fraction result from the VBE method, the gas void fraction was calculated using the pressure drop across a pair of downhole pressure gauges (*P*_*i*_ and *P*_*j*_). As shown in Eq. (6), the pressure drop is a function of the gas void fraction as

8 $${\Delta P}_{ij}=0.052 *\alpha \left({\rho }_{w}-{\rho }_{g}\right) *h$$

$$\alpha =\frac{{\Delta P}_{ij}}{0.052 * \left({\rho }_{w}-{\rho }_{g}\right) *h}$$

where, *h* is the gas–water mixture height, ${\rho }_{w}$ and ${\rho }_{g}$ are water and gas densities, respectively, $\alpha$ is the gas void fraction to be calculated, and 0.052 is the unit conversion factor when the units of the pressure, density, and height are expressed in psi, pounds per gallon, and ft, respectively. Figure 7 illustrates the application of the pressure drop method for the same data as used in Case Study 1, when the gas–water mixture region is between the gauges at 2023 ft (P_2_) and 3502 ft (P_3_). The change in the pressure drop between the two gauges as the gas slug migrates through this region (ΔP_23_) can be estimated from the gauge data shown in Fig. 7. The gas–water mixture height can be accurately estimated using the VBE method, as discussed in “Case study 1: gas–liquid interface tracking” section. Using Eq. (6), the gas void fraction in the gas–water region is estimated as 0.06, which shows good agreement with the estimation based on the VBE workflow [Fig. 6 and Eq. (5)].

For further validation, the gas fraction results from the VBE workflow and the pressure difference approach were compared at two other locations as the gas migrates through the annulus. The results summarized in Table 2 show good agreement between the two approaches rendering confidence in the results obtained from the novel VBE workflow.

**Table 2:** Gas void fractions estimated using the VBE and pressure difference approach.

Gas location	Pressure difference method			VBE method	
	Gas–water mixture height, *h* (ft)	Pressure difference, *ΔP*_*ij*_ (psi)	Gas fraction, ${\alpha }_{g}$	Speed of sound, *c*_*m*_ (ft/s)	Gas fraction, ${\alpha }_{g}$
Between 487 and 2023 ft	740	22.42	0.06	1310	0.05
Between 2023 and 3502 ft	774	19.78	0.07	1680	0.06
Between 3502 and 5025 ft	1058	25.18	0.08	1750	0.07

## Conclusion

This study presents a novel workflow to analyze fiber-optic DAS data that takes into account the speed of sound of a certain phase to isolate the acoustic energy or signal contributed by that phase in a multiphase medium. The VBE workflow involves a series of signal processing and conditioning steps aimed at reducing background noise and enhancing the signal of interest. The VBE workflow was successfully demonstrated using a dataset acquired in a 5163-ft-deep wellbore, for estimating gas void fraction and real-time gas–liquid interface tracking. Multiphase flow characterization has many practical applications, especially in the wellbore flow setting evaluated in this study. Interface tracking is crucial for estimating oil–water and gas–water contacts in the reservoir, estimating gas influx volumes, and diagnosing liquid above the pump. The gas fraction is one of the most fundamental parameters required in different flow calculations, such as two-phase viscosity, density, and pressure drop calculations. The gas fraction results from the VBE workflow were validated with downhole gauge data which shows good agreement.

We note that the determination of gas fraction by speed-of-sound is highly nonlinear and double valued (an asymmetric “bathtub” curve). However, a virtual flow meter that provides a distributed gas-fraction for any specific wellbore can be defined by (a) combining measurements of the mixing length (Case Study 1), (b) temperature and pressure estimates (e.g., from gauges at the top and bottom of the well via a multiphase flow model), and (c) bulk flow rates (e.g., from surface metering).

## Data Availability

The datasets generated and/or analyzed during the current study are included in this published article or are available from the corresponding author upon reasonable request.
